# Genetic Characterization of a Novel Bovine Rotavirus A G37P[52] Closely Related to Human Strains

**DOI:** 10.3389/fvets.2022.931477

**Published:** 2022-07-14

**Authors:** Vikash K. Singh, Victor Neira, Barbara Brito, Naomi Ariyama, Matt Sturos, Sunil K. Mor

**Affiliations:** ^1^Veterinary Diagnostic Laboratory, Department of Veterinary Population Medicine, College of Veterinary Medicine, University of Minnesota, St. Paul, MN, United States; ^2^Departamento de Medicina Preventiva Animal, Facultad de Ciencias Veterinarias y Pecuarias, Universidad de Chile, Santiago, Chile; ^3^The iThree Institute, University of Technology Sydney, Sydney, NSW, Australia

**Keywords:** rotavirus A, novel genotype, G37P[52], emerging virus, bovine, calf diarrhea

## Abstract

Bovine rotavirus A (boRVA) strains are common causative agents of diarrhea in calves, resulting in economic losses to the beef and dairy industry. Importantly, this virus has a zoonotic relevance due to its ability to reassort with human rotaviruses. In this study, fecal samples were collected from three calves with diarrhea during an outbreak on a dairy farm. The genetic material of boRVA was detected by real-time reverse transcription PCR (rtPCR) in two samples. Then the virus in one of these positive samples was identified as a novel boRVA genotype closely related with human rotavirus strains mainly from the USA based on whole-genome characterization. However, we consider the novel boRVA as the etiological agent of the outbreak due to the lesions associated with a rotavirus infection. Further studies are necessary to clarify the evolutionary advantages that novel rotavirus genotypes may have.

## Introduction

Rotaviruses cause neonatal diarrhea in mammals and avian species. The rotavirus genome consists of 11 double-stranded RNA segments, containing six structural viral protein genes (VP1-VP4, VP6, and VP7) and five non-structural protein genes (NSP1–NSP5/NSP6) (1). So far, ten rotavirus species (A-J) have been proposed based on genetic and antigenic differences on the middle capsid layer protein gene (VP6) (2). Rotavirus A (RVA) strains are primarily classified into genotypes/serotypes based on their outer capsid proteins, VP7 (G type, for glycoprotein) and VP4 (P type, for protease sensitive), with a high genetic and antigenic diversities. Before this detection, 36 G and 51 P genotypes have been identified. To better describe the RVA viral diversity, a genotyping method that includes all segments has been established using the following nomenclature: Gx(VP7), P[x](VP4), Ix(VP6), Rx(VP1), Cx(VP2), Mx(VP3), Ax(NSP1), Nx(NSP2), Tx(NSP3), Ex(NSP4), and Hx(NSP5) (1).

Bovine RVA (boRVA) strains are a typical cause of neonatal diarrhea in calves but can also cause disease in adults. Calf diarrhea is associated with a significant economic loss for the cattle industry (3, 4). The most frequent boRVA genotypes are G4-G6-G8-G10-G12 and P[1]-P[5]-P[11] (4). The boRVA strains containing G6P[5], G6P[11], G10P[5], and G10P[11] genotypes are the most prevalent worldwide (5). Mixed infections of boRVA are common, and the virus can also be found in healthy animals (6). In 2011, two new genotypes—G21P[29] and G24P[33]—were described from asymptomatic cattle, highlighting the importance of bovines as reservoirs of novel RVA (7). Interestingly, reassortant boRVA strains can contain RVA segments from swine and humans (6). Furthermore, boRVA strains contributed to novel human strain genesis on several occasions, suggesting its relevance as a zoonotic pathogen (8). In this study, we report a novel RVA genotype associated with a case of enteric disease in calves in Minnesota, USA.

## Materials and Methods

### Samples

In September 2018, ±10-day-old calves on a dairy farm developed diarrhea and became dehydrated. Fecal samples from three 14-day-old cross-bred dairy symptomatic calves were submitted to the Minnesota Veterinary Diagnostic Laboratory (MVDL), College of Veterinary Medicine, University of Minnesota, for diagnosis.

### Diagnostic Tests

The complete laboratory diagnostic investigation included aerobic, anaerobic, and *Salmonella* spp. Culture; fecal flotation; immunofluorescent antibody testing for *Cryptosporidium* spp. and *Giardia* spp.; and real-time PCR testing for RVA, rotavirus B, and bovine coronavirus. RVA genetic material was detected in two of three fecal samples with one sample strongly positive with a PCR cycle threshold (Ct) of 18.11. The partial VP4 and VP7 gene sequences were obtained by Sanger sequencing of this strong positive sample.

### Whole-Genome Sequencing

A next-generation sequencing (NGS) was performed on the RT-PCR-strongly positive fecal sample to obtain the whole genome sequence of the detected RVA. The sample was sequenced using an Illumina MiSeq with a V2 sequencing kit, 250-bp paired-end reads, and NuGen Trio RNA library preparation kit. The FASTQ files were analyzed using an in-house bioinformatics pipeline for trimming to remove Illumina adapters using Trimmomatic (v 0.39) with a minimum quality score of 20 (9). Then, host contamination was removed using Bowtie2 v 2.4.4 (10). The SPAdes v3.15.2 with k-mer values of 21, 31, 41, 51, 61, and 71 and the options careful was used for assembly of unmapped reads (11). Extracted contigs were analyzed using BLASTx at NCBI to determine the taxonomy.

### Viral Segment Genotyping

Genotypes for all complete segment sequences were determined using the Virus Pathogen Database and Analysis Resource (ViPR) Rotavirus A Genotype Determination (12). Segments that did not meet the inclusion criteria to any existing genotype were submitted to the Rotavirus Classification Working Group (RCWG) for validation as new genotypes (13).

### Phylogenetic Analysis

The phylogeny was constructed for all segments, using reference sequences for each genotype. These sequences were obtained from the NCBI Virus Variation resource and aligned with the novel RVA sequences (12). Finally, phylogenetic trees were inferred by maximum likelihood using the GTR+R model, and the bootstrap values were determined by 1,000 replicates. The complete genome of RVA named RVA-BosTaurus-MN41364-2018-G37P-52 was assembled and deposited in GenBank (Accession numbers MW752838-MW752848); details on Supplementary Table 1.

## Results and Discussion

The partial VP4 and VP7 gene sequences obtained by the Sanger sequencing of the strongest RVA positive sample had a low nucleotide identity compared to reference strains. To further characterize this virus, NGS was conducted with a total of 97,420 viral reads obtained, of which 23,753 were reads related to the novel RVA. Other viruses such as astrovirus and norovirus were also detected. However, these last two viruses have an unclear clinical significance (14). The RVA was further genetically characterized by analyzing all viral segments separately (Genbank Accession numbers MW752838-MW752848). First, the NCBI BLAST analysis confirmed the viral identity, and in general hits with other human or bovine RVA segments ranged between 76.1 and 99.3% of identity (Table 1). Nine out of 11 segments were successfully identified as I2-R2-C2-M2-A3-N2-T9-E2-H3. However, VP7 and VP4 did not meet the cutoff threshold for any previously described genotype, indicating potential novel G and P genotypes (Supplementary Figure 1). Accordingly, segments VP7 and VP4 were submitted to the RCWG for validation as new genotypes (13). Thus, RCWG confirmed the new genotypes which were assigned as G37 and P[52] (Supplementary Informations 1, 2).

**Table 1:** Novel bovine rotavirus A genotyping and strains with the highest nucleotide identity.

**Genotype**	**Gene Segment**	**Product description**	**Closest strain**	**Identity (%)**
G37^#^	VP7	Glycoprotein	KC895768|Bos_taurus|Argentina|2005	76.86
P[52]^#^	VP4	Protease sensitive	AB055967.1|Capra_hircus|Korea|1998	78.57
I2	VP6	Intermediate capsid shell	AB573073|Bos_taurus|Japan|2008/06	96.06
R2	VP1	RNA dependent RNA polymerase	DQ870493|Bos_taurus|USA|	95.01
C2	VP2	Core shell protein	KT281121|Homo_sapiens|USA|2012	97.24
M2	VP3	Methyltransferase	LC340015|Homo_sapiens|Japan|2014	95.61
A3	NSP1	Interferon Antagonist	FJ422135|Macaca_nemestrina|USA|	98.17
N2	NSP2	NTPase	EU636931|rhesus_monkey|USA|1980	97.38
T9	NSP3	Translation enhancer	KT281126|Homo_sapiens|USA|2012	97.26
E2	NSP4	Enterotoxin	JN248456|Bos_taurus|Denmark|2007	95.45
H3	NSP5	Phosphoprotein	KT281130|Homo_sapiens|USA|2012	98.79

# *Novel genotypes*.

**Figure 1:**
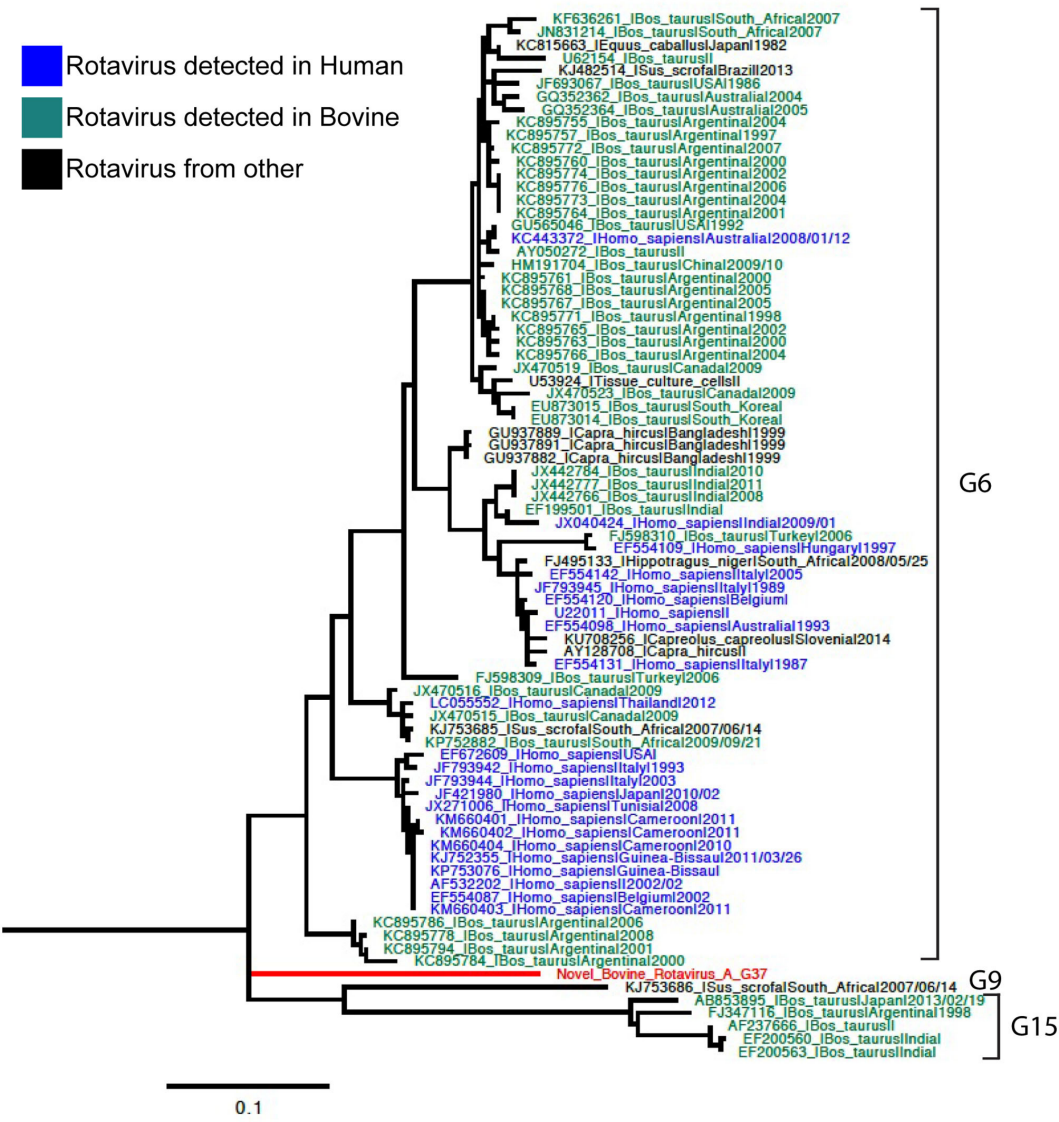
Phylogenetic tree of VP7 rotavirus A (RVA) segment using ML GTR+R 1,000 replicates. Reference sequences are depicted in blue for human origin rotaviruses, green for bovine origin rotaviruses, and orange for swine origin rotaviruses. Novel rotavirus sequence is depicted in red.

**Figure 2:**
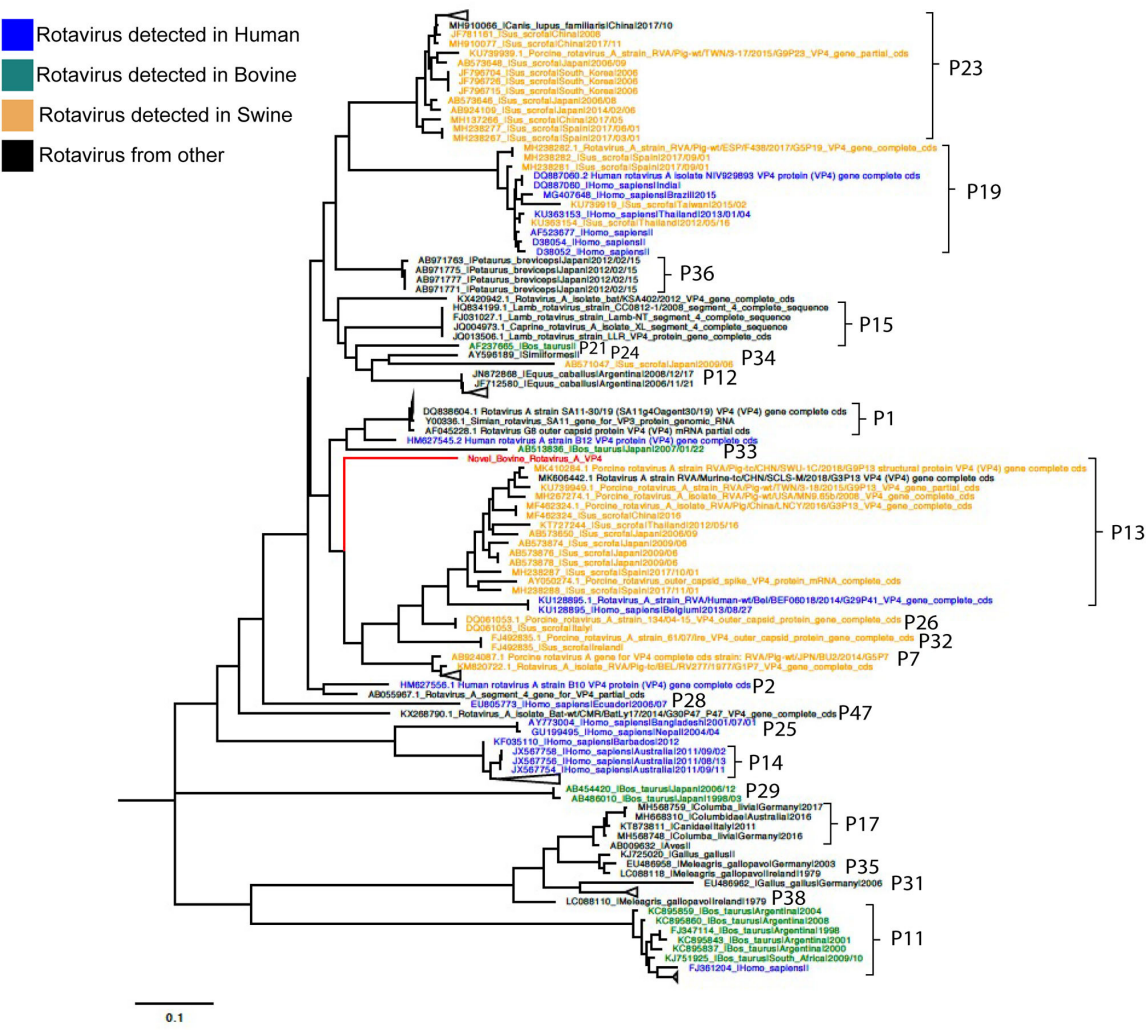
Phylogenetic tree of VP4 RVA segment using ML GTR+R 1,000 replicates. Reference sequences are depicted in blue for human origin rotaviruses, green for bovine origin rotaviruses, and orange for swine origin rotaviruses. Novel rotavirus sequence is depicted in red.

Phylogenetic analyses for VP7 segment showed the novel G37 as a singleton most closely grouped with G15, G9, and G6 genotypes usually found in bovines and humans (Figure 1). The VP4 segment phylogeny depicts the novel P[52] as a singleton, but clustering with P[7], P[13], P[26], P[32], and P[41], which are genotypes usually isolated in pigs and humans (Figure 2). The remaining segments showed a close relation with human and bovine strains obtained mainly from the USA and Japan, respectively (Supplementary Figures 2–10).

Genotyping was achieved based on all viral genome segments resulting in a novel G37 and P[52] types, genotype assigned by the RCWG. Phylogenetic analyses showed that this virus of bovine- and swine-origin RVA is related to human genotypes. Concerning the I2-R2-C2-M2-A3-N2-T9-E2-H3 genotypes, these also related primarily to human and bovine viruses. These results agree with previous studies that suggest a complex interaction and prevalent reassortment events among animal and human RVA (8, 15, 16), therefore, emphasizing the RVA zoonotic potential.

Additionally, *Escherichia coli* was detected in one and *Clostridium perfringens* in the three samples analyzed in this study. Both bacteria are considered enteric pathogens in neonatal calves, specifically some subtypes such as *E. coli* F5 (K99+) and *C. perfringens* type C (17, 18). However, the bacteria isolated in this study were not subtyped. On the other hand, RVA has a well-studied primary role in calf diarrhea (4, 14). Therefore, we consider that the novel boRVA was involved in the enteric disease concomitant with *E. coli* and *C. perfringens*. Other enteric pathogens such as *Salmonella* spp., *Cryptosporidium* spp., and bovine coronavirus were not detected.

We identified a novel boRVA associated with calf diarrhea and closely related to human rotavirus strains. Our results highlight the efficiency of NGS as a high-resolution tool for genetic characterization of emerging viral pathogens in cattle. Further, whole-genome sequence-based characterization is essential to understand the evolution of rapidly evolving divergent viruses such as RVA. Further studies are necessary to clarify the evolutionary advantages that novel genotypes may have.

## Data Availability Statement

The datasets presented in this study can be found in online repositories. The names of the repository/repositories and accession number(s) can be found in the article/Supplementary Material.

## Ethics Statement

Ethical review and approval was not required for the animal study because this study corresponds to a case study of a deceased farm animal; therefore, bioethical approval was not obtained.

## Author Contributions

SM and VN: study design and conceptualization, funding, and resources. MS: sample collection and processing. MS, VS, and SM: performed the assays. BB, VS, NA, and VN: data analysis. VS, NA, BB, VN, and SM: wrote the paper. All authors critically evaluated the paper and contributed to the article and approved the submitted version.

## Funding

This study was partially funded by the Molecular Development Lab, Veterinary Diagnostic Lab, College of Veterinary Medicine, University of Minnesota. VN and NA are funded by Programa Fondecyt No. 1211517.

## Conflict of Interest

The authors declare that the research was conducted in the absence of any commercial or financial relationships that could be construed as a potential conflict of interest.

## Publisher's Note

All claims expressed in this article are solely those of the authors and do not necessarily represent those of their affiliated organizations, or those of the publisher, the editors and the reviewers. Any product that may be evaluated in this article, or claim that may be made by its manufacturer, is not guaranteed or endorsed by the publisher.
