# Supplementary material for: Genetic Characterization of a Novel Bovine Rotavirus A G37P[52] Closely Related to Human Strains
Source: Front Vet Sci. 2022 Jul 14;9:931477. doi: 10.3389/fvets.2022.931477 (PMC9330215; doi:10.3389/fvets.2022.931477)
Supplement: Supplementary file 1 [file Data_Sheet_1.docx]

Supplementary Material

**Supplementary Table 1**. GenBank accession numbers.

| Sequence Name | GenBank accession number |
| --- | --- |
| RVA-BosTaurus-MN41364-2018-G37P-52_VP1 | MW752838 |
| RVA-BosTaurus-MN41364-2018-G37P-52_VP2 | MW752839 |
| RVA-BosTaurus-MN41364-2018-G37P-52_VP3 | MW752840 |
| RVA-BosTaurus-MN41364-2018-G37P-52_VP4 | MW752841 |
| RVA-BosTaurus-MN41364-2018-G37P-52_NSP1 | MW752842 |
| RVA-BosTaurus-MN41364-2018-G37P-52_VP6 | MW752843 |
| RVA-BosTaurus-MN41364-2018-G37P-52_NSP3 | MW752844 |
| RVA-BosTaurus-MN41364-2018-G37P-52_NSP2 | MW752845 |
| RVA-BosTaurus-MN41364-2018-G37P-52_VP7 | MW752846 |
| RVA-BosTaurus-MN41364-2018-G37P-52_NSP4 | MW752847 |
| RVA-BosTaurus-MN41364-2018-G37P-52_NSP56 | MW752848 |

**Supplementary information 1**. RCWG notification for novel VP4 genotype P[52]

**Supplementary information 2**. RCWG notification for novel VP7 genotype G37

**Supplementary Figure 1**. Trees obtained from Rota C analysis of VP7 (left) and VP4 (right) segments, sequences from this study labeled with red dots.

**Supplementary Figure 2**. Phylogenetic tree of VP1 RVA segment using ML GTR+R 1000 replicates.

**Supplementary Figure 3**. Phylogenetic tree of VP2 RVA segment using ML GTR+R 1000 replicates.

**Supplementary Figure 4**. Phylogenetic tree of VP3 RVA segment using ML GTR+R 1000 replicates.

**Supplementary Figure 5**. Phylogenetic tree of VP6 RVA segment using ML GTR+R 1000 replicates.

**Supplementary Figure 6**. Phylogenetic tree of NSP1 RVA segment using ML GTR+R 1000 replicates.

**Supplementary Figure 7**. Phylogenetic tree of NSP2 RVA segment using ML GTR+R 1000 replicates.

**Supplementary Figure 8**. Phylogenetic tree of NSP3 RVA segment using ML GTR+R 1000 replicates.

**Supplementary Figure 9**. Phylogenetic tree of NSP4 RVA segment using ML GTR+R 1000 replicates.

**Supplementary Figure 10**. Phylogenetic tree of NSP5/6 RVA segment using ML GTR+R 1000 replicates.
